# A Detailed Flow Cytometric Analysis of Immune Activity Profiles in Molecular Subtypes of Colorectal Cancer

**DOI:** 10.3390/cancers12113440

**Published:** 2020-11-19

**Authors:** Xingru Li, Agnes Ling, Therese G. Kellgren, Marie Lundholm, Anna Löfgren-Burström, Carl Zingmark, Martin Rutegård, Ingrid Ljuslinder, Richard Palmqvist, Sofia Edin

**Affiliations:** 1 Department of Medical Biosciences, Pathology, Umeå University, 90185 Umeå, Sweden; xingru.li@umu.se (X.L.); agnes.ling@umu.se (A.L.); therese.kellgren@umu.se (T.G.K.); marie.lundholm@umu.se (M.L.); anna.lofgren-burstrom@umu.se (A.L.-B.); carl.zingmark@umu.se (C.Z.); richard.palmqvist@umu.se (R.P.); 2 Department of Surgical and Perioperative Sciences, Surgery, Umeå University, 90185 Umeå, Sweden; martin.rutegard@umu.se; 3 Wallenberg Centre for Molecular Medicine, Umeå University, 90187 Umeå, Sweden; 4 Department of Radiation Sciences, Oncology, Umeå University, 90185 Umeå, Sweden; ingrid.ljuslinder@umu.se

**Keywords:** colorectal cancer, immune activity profile, microsatellite instability, consensus molecular subtypes

## Abstract

**Simple Summary:**

Colorectal cancer is one of the deadliest cancers worldwide, with around 40% of patients dying from distant metastasis. Tumour immune cell infiltration has powerful positive prognostic value in this disease, suggesting immunotherapy as a potential treatment modality. The aim of this explorative study was to assess in detail the local and systemic immune response in different molecular subgroups of colorectal cancer. An improved molecular understanding of the disease may lead to important advances in personalised medicine, identifying prognostic and predictive tools, in addition to new therapeutic targets.

**Abstract:**

The local anti-tumour immune response has important prognostic value in colorectal cancer (CRC). In the era of immunotherapy, a better understanding of the immune response in molecular subgroups of CRC may lead to significant advances in personalised medicine. On this note, microsatellite instable (MSI) tumours have been characterised by increased immune infiltration, suggesting MSI as a marker for immune inhibitor checkpoint therapy. Here, we used flow cytometry to perform a comprehensive analysis of immune activity profiles in tumour tissues, adjacent non-malignant tissues and blood, from a cohort of 69 CRC patients. We found several signs of immune suppression in tumours compared to adjacent non-malignant tissues, including T cells more often expressing the immune checkpoint molecules programmed cell death protein (PD-1) and cytotoxic T lymphocyte-associated protein 4 (CTLA-4). We further analysed immune cell infiltration in molecular subgroups of CRC. MSI tumours were indeed found to be associated with increased immune infiltration, including increased fractions of PD-1^+^ T cells. No correlation was, however, found between MSI and the fraction of CTLA-4^+^ T cells. Interestingly, within the group of patients with microsatellite stable (MSS) tumours, some also presented with increased immune infiltration, including comparably high portions of PD-1^+^ T cells, but also CTLA-4^+^ T cells. Furthermore, no correlation was found between PD-1^+^ and CTLA-4^+^ T cells, suggesting that different tumours may, to some extent, be regulated by different immune checkpoints. We further evaluated the distribution of immune activity profiles in the consensus molecular subtypes of CRC. In conclusion, our findings suggest that different immune checkpoint inhibitors may be beneficial for selected CRC patients irrespective of MSI status. Improved predictive tools are required to identify these patients.

## 1. Introduction

Colorectal cancer (CRC) is one of the deadliest cancers worldwide [1]. Despite medical advances, still around 40% of the patients will die as the result of distant metastasis. The immune response has powerful prognostic value in CRC [2], making CRC patients promising candidates for immunotherapy. However, with this promise also comes higher demands on prognostic and predictive tools for individualised treatment plans. 

The positive prognostic importance of T lymphocytes in CRC has been widely accepted, and has subsequently led to a joint task force to introduce an immunohistochemical evaluation of T cell markers, the Immunoscore, into clinical practice [3]. Because of the powerful prognostic role of immune cells in CRC, there is hope that immunotherapy in CRC will evolve into a new and promising treatment frontier. Many therapies aiming at modulating the immune response are currently under development. Expansion of cytotoxic T cells, for example, is negatively regulated by the immune checkpoint molecules, cytotoxic T lymphocyte-associated protein 4 (CTLA-4) and programmed cell death protein (PD-1/PDCD-1) [4]. Targeted therapy against CTLA-4 (ipilimumab, tremelimumab) and PD-1 (nivolumab, pembrolizumab) has been FDA approved, showing promising results in many types of cancer including melanoma, lung cancer and head and neck cancer [5]. The clinical response to immune checkpoint inhibitors by the majority of patients with metastatic CRC has, however, been found wanting [6,7]. In addition to immune checkpoint inhibitors, other immunotherapies such as tumour vaccines also demonstrate interesting results [8]. These therapeutic advancements will nonetheless place higher demands on the molecular understanding of this disease, including prognostic and predictive tools, in order to select patients who may benefit from treatment. 

CRC is a heterogeneous disease that develops through different molecular pathways, three of which have been well described [9]: The classical microsatellite stable (MSS) adenoma to carcinoma pathway [10], which is responsible for approximately 85% of CRCs, the microsatellite instability (MSI) pathway [11,12] and the CpG island methylator phenotype (CIMP) [13]. An association has been shown between MSI and an increased infiltration rate of immune cells, which might in part explain the better outcome in this patient group [14,15]. During recent years, MSI has emerged as a major predictive marker for immune checkpoint inhibitor therapy; pembrolizumab or nivolumab, alone or in combination with ipilimumab, have been FDA approved for chemoresistant CRC patients with metastatic MSI tumours [16]. However, the majority of CRCs are MSS, and an increased understanding of the heterogeneous immunity of the MSS tumours may identify patients who could potentially respond to immune therapy. Indeed, increased immune infiltration gives a prognostic advantage also in patients with MSS tumours [17].

In recent years, the molecular understanding of CRC has gradually improved. Large scale data sharing and transcriptomics analyses by an international consortium have led to the identification of four consensus molecular subtypes (CMSs) of CRC: CMS1 (hypermutated, MSI, *BRAF* mutation, CIMP high, immune activation), CMS2 (epithelial, marked WNT and MYC signalling activation), CMS3 (epithelial and evident metabolic dysregulation, *KRAS* mutation) and CMS4 (mesenchymal, prominent transforming growth factor-β (TGF-β) activation, stromal invasion and angiogenesis) [18]. Currently, the CMS groups are suggested as the most extended classification system available for CRC, in terms of biological and clinical behaviour. 

Here, we performed a detailed analysis of immune activity profiles in tumour tissue, adjacent non-malignant tissue, and blood from a cohort of CRC patients using flow cytometry. The aim was to better characterise the local and systemic immune response in relation to clinical, pathological and molecular characteristics of CRC. An increased understanding of the structure and organisation of the immune response in molecular subgroups of CRC may identify important predictive and prognostic tools as well as new targets for therapy.

## 2. Results

### 2.1. Immune Profiles and Patient Characteristics

We have performed a detailed analysis of immune profiles in a cohort of 69 CRC patients. Immune profiles were analysed by flow cytometry in immune cells extracted from CRC tumour tissues, adjacent non-malignant tissues, as well as blood. The immune profiles included analyses of T lymphocytes (CD4^+^ or CD8^+^), B lymphocytes (CD19^+^), natural killer (NK) cells (CD56^+^/CD16^+^) and monocytes/macrophages (CD14^+^), along with markers for their activation or inhibition. The clinicopathological and molecular characteristics of the study patients can be found in Table 1. In brief, the majority of patients were diagnosed with Stage II–III tumours (77%) and a quite large fraction of the tumours (54%) were localised to the right colon (including transverse colon). Accordingly, *BRAF* mutated tumours (34%) and tumours, classified as MSI (24%) and CMS1 (37%), were also slightly over-represented. The CMS4 group was instead slightly under-represented (9%) [18]. 

No significant correlations were found for immune cell subsets in tumour tissue to age, gender, localisation, tumour grade or tumour type (mucinous/non-mucinous) in this study. A weak correlation of an increased fraction of T-helper (Th) cells expressing PD-1 (*p* = 0.017), and cytotoxic T (Tcyt) cells expressing PD-1 (*p* = 0.053), to lower tumour stages was found. Tumour stage was also weakly correlated to the fraction of NK cells expressing CD69 (*p* = 0.025), with an increased fraction found in tumours of higher stages.

### 2.2. Immune Profiles in CRC Show Signs of Immune Suppression

**Figure 1:**
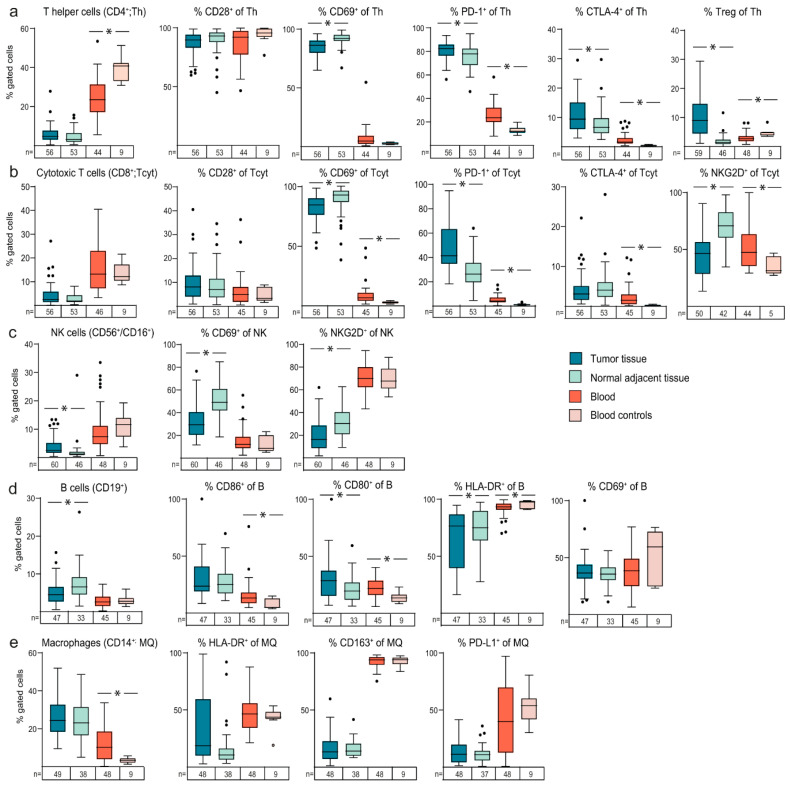
Distribution of immune profiles in tissue and blood of patients diagnosed with colorectal cancer (CRC). Box and whiskers plots illustrating percentages of subsets of (**a**) T helper cells, (**b**) cytotoxic T cells, (**c**) NK cells, (**d**) B cells and (**e**) macrophages, in isolated mononuclear immune cells from tumour tissue compared to adjacent normal tissues, and blood samples from CRC patients compared to controls, as indicated. Horizontal lines indicate median values. * indicates *p*-values < 0.05.

Immune profiles were compared between tumour tissues, non-malignant adjacent tissues and blood. Tumours were generally found to be marginally more infiltrated by immune cells than their adjacent normal colorectal epithelial tissues (Figure 1). No significant differences were found for the CD4^+^ Th cells and the CD8^+^ Tcyt cells between tumour and normal tissue, but when analysing markers of immune activation or inhibition in these T cells subsets, we found several signs of immune suppression in tumour tissue compared to normal adjacent tissue. For example, the Th cells and the Tcyt cells in tumour tissue expressed the activation markers CD69 less often, and more often expressed the inhibitor PD-1, than those in adjacent normal tissue (Figure 1a,b). In addition, Tcyt cells in tumour tissue expressed the activation marker natural killer group 2D (NKG2D) less often, while Th cells in tumour tissue were positive more often for the inhibitor CTLA-4 (Figure 1a,b). The fraction of Th cells defined as T regulatory cells (Tregs) was also significantly increased in tumour tissue compared to normal adjacent tissue (Figure 1a). These results support that there are plausible targets for treatment with an intent to reactivate immunity (using e.g., PD-1 and CTLA-4 immune checkpoint inhibitors). The increased fraction of T cells expressing PD-1 or CTLA-4 in tumour compared to normal adjacent tissue was further mirrored in blood from CRC patients compared to healthy controls (Figure 1a,b). On the contrary, the increased fraction of Th cells defined as Tregs in tumour compared to normal tissue was instead paralleled by a decreased fraction of these cells in blood of CRC patients compared to controls, and the decreased fractions of T cells expressing CD69 or NKG2D in tumour compared to normal tissue, was paralleled by an increased fraction of these cell subsets in blood of CRC patients compared to controls (Figure 1a,b). 

NK cells (CD56^+^/CD16^+^) were found to be significantly enriched in tumours compared to adjacent normal tissues, and to express CD69 and NKG2D less often (Figure 1c). B cells (CD19^+^) were found infiltrating some CRC tumours, but the levels of B cells were however slightly lower in tumour compared to adjacent normal tissues, with slightly increased expression of CD80 and decreased expression of the antigen presenting molecule human leukocyte antigen-DR (HLA-DR) (Figure 1d). No significant differences were found for CD14^+^ macrophages in tumour compared to normal adjacent tissues (Figure 1e).

### 2.3. The Relation of Immune Profiles to Molecular Subtypes of CRC

**Figure 2:**
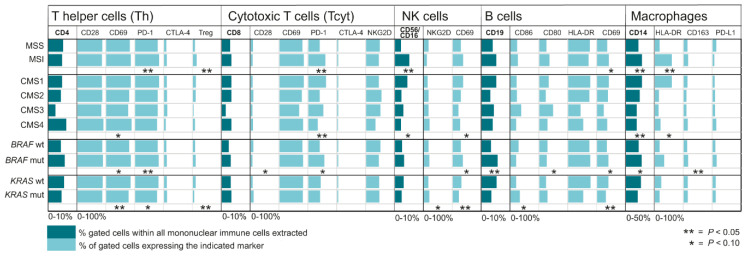
Shown is median percentage of gated cell populations in mononuclear immune cells (in dark blue) isolated from tumour tissue of patients with colorectal cancer, and percentage of these gated cells expressing the indicated markers (in light blue). * indicate *p*-values < 0.10 and ** indicate *p*-values < 0.05.

In an attempt to improve the understanding of immune activity in molecular subgroups of CRC, we analysed the distribution of different immune cell subsets in relation to tumour MSI/MSS status, CMS subtype, as well as *BRAF* and *KRAS* mutation status, and identified several significant relations (Figure 2). MSI tumours were found to be positively associated with an increased fraction of T cells expressing PD-1 (both Th and Tcyt subsets) and Th cells defined as Tregs (Figure 2). However, no correlation was found between MSI tumours and the fraction of T cells expressing CTLA-4. NK cells were also significantly enriched in MSI tumours, along with macrophages and the fraction of HLA-DR^+^ M1 macrophages, supporting the view of MSI tumours as highly immunogenic. Instead turning our attention to tumour CMS status and the correlation to immune markers, we found significant correlations of CMS status to PD-1^+^ Tcyt cells as well as macrophages (Figure 2). *BRAF* mutated tumours showed higher fractions of PD-1^+^ T cells, B cells and CD163^+^ macrophages compared to *BRAF* wild type tumours (Figure 2). On the other hand, *KRAS* mutated tumours were associated with decreased fractions of CD69^+^ Th cells, Tregs, CD69^+^ NK cells and CD69^+^ B cells (Figure 2). 

**Figure 3:**
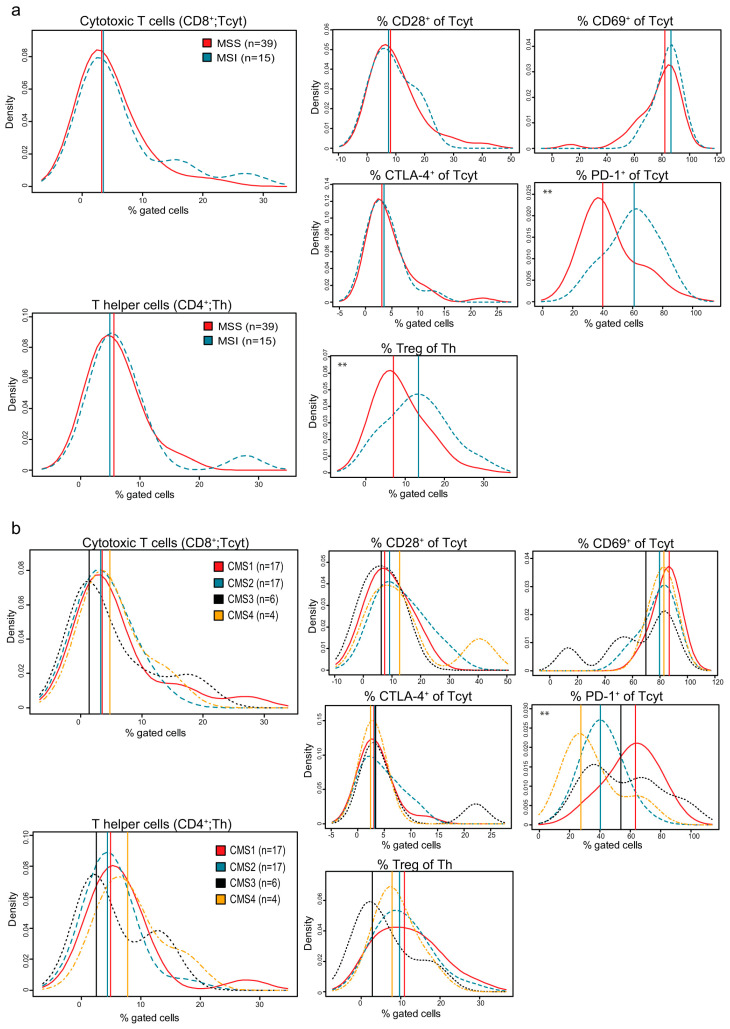
Density plots illustrating the distribution of indicated immune markers predicted by (**a**) MSI/MSS subgroup and (**b**) CMS subgroup. Vertical lines indicate median values. ** indicates *p*-values < 0.05.

Interestingly, by stratifying the immune checkpoint molecules, PD-1 and CTLA-4, on T cells by MSI/MSS status, we found fractions of PD-1^+^ Tcyt cells (Figure 3a) and also PD-1^+^ Th cells (Supplementary Figure S1a) in some MSS tumours comparable to those in MSI tumours, suggesting there is a similar immune suppression in selected MSS tumours. A few MSS tumours also had increased fractions of CTLA-4^+^ T cyt cells (Figure 3a). However, we found no positive correlation between PD-1^+^ and CTLA-4^+^ T cells (for Th, r_s_ = 0.022, *p* = 0.870 and for Tcyt, r_s_ = −0.172, *p* = 0.206), suggesting that PD-1 and CTLA-4 may define different populations of T cells and different tumours. In addition, some MSS tumours showed relatively high fractions of Th cells defined as Tregs, as well as NK cells and fractions of HLA-DR^+^ M1 macrophages (Figure 3a and Supplementary Figure S1b,d). These findings suggest that some MSS tumours share a similar immune activity profile to those of MSI tumours.

Analysing the distribution of the immune checkpoint molecules in the different CMS subgroups more closely further revealed an increased fraction of Tcyt cells expressing PD-1 in CMS1 tumours, but also in selected CMS3 and CMS4 tumours, while increased fractions of Tcyt cells expressing CTLA-4 was found mainly in selected CMS3 tumours (Figure 3b). Macrophages were found to be enriched in CMS1 tumours, but also in selected CMS3 tumours (Supplementary Figure S2d). Similar fractions of HLA-DR^+^ M1 macrophages could be found in CMS1 tumours, as well as in some CMS3 and CMS4 tumours, but were reduced in CMS2 tumours. High fractions of macrophages expressing PD-L1 were found mainly in CMS1 tumours (Supplementary Figure S2d). The distribution of the remaining immune markers according to CMS status can be found in Figure 3 and Supplementary Figure S2. All in all, immune activity profiles were found linked mainly to CMS1 tumours, but similar immune activity profiles could however also be found in other tumour subgroups. 

### 2.4. The Local Tumour Immune Response in Relation to Systemic Immunity

A few of the findings of increased fractions of certain immune cells subtypes in tumour compared to adjacent normal tissue described in Figure 1 were paralleled by a comparable change in blood from CRC patients compared to controls. To analyse how the local anti-tumour immune response is reflected in systemic immunity, we further studied the correlations between tumour infiltrating and systemic immune cells. Analysing one immune parameter at a time, a positive correlation was found for only three of the markers: CD69^+^ Th cells, CD28^+^ Tcyt cells and NKG2D^+^ Tcyt cells (Table 2). However, of these, the fraction of NKG2D^+^ Tcyt cells was found decreased in tumour tissue compared to normal adjacent tissue, but was paralleled by an increase in blood of CRC patients compared to controls (Figure 1). Comparisons of the correlations between all immune parameters in tumour tissue and blood did not show a strong overall relationship (Supplementary Table S1), suggesting that the cellular immune profile in blood might not generally be a good mirror of the local anti-tumour immune response.

## 3. Discussion

In the present study, we used detailed flow cytometry analyses to evaluate immune activity profiles in molecular subtypes of colorectal cancer. By comparing tumour tissues to adjacent non-malignant tissues, we found several signs of immune suppression in the tumours. These changes included decreased fractions of T cells expressing the activation markers CD69 and NKG2D, and increased fractions of T cells expressing the immune inhibitory checkpoint molecules PD-1 and CTLA-4, together with an increased fraction of T regulatory cells. To add, infiltrating NK cells expressed less of the activation markers. These results show that treatment with an intent to reactivate immunity using e.g., immune checkpoint inhibitors may have potential for some CRC patients. Accurate predictive tools will be essential to identify these patients.

We next evaluated the immune activity profiles in relation to tumour molecular subtypes. MSI tumours are shown to have a high neoantigen load and lymphocytic infiltration, as the result of a high tumour mutational burden [19]. This, together with the proven benefits of immune checkpoint inhibitor therapy in patients with MSI tumours [7], has focused immune related research in CRC on this tumour subtype. However, it is well known that not all MSI tumours are highly immune infiltrated, and that some MSS tumours also display a strong immune response [17]. Furthermore, immune infiltration has been shown to be a positive prognostic factor in both MSI and MSS subgroups [17]. In our study, we indeed found that MSI tumours were highly and positively associated with the fraction of PD-1^+^ T cells, Tregs, NK cells and M1 macrophages, supporting the general view of MSI tumours as highly immunogenic. As this is in line with findings previously reported in literature, the methodology behind this work is corroborated. An increased number of infiltrating PD-1^+^ lymphocytes in CRC MSI tumours compared to MSS tumours has previously been shown [20,21]. Lee et al. found high PD-1 expression on immune cells in 50% of MSI compared to 13% of MSS tumours. Ahtiainen et al. showed PD-1 high immune cells in 81% of MSI versus 49% of MSS tumours. Similar to these studies, we found that not all MSI tumours had an increased fraction of PD-1^+^ T cells. Furthermore, we did find that some patients with MSS tumours presented with a similar immune activity profile as those with MSI tumours, including comparably high portions of PD-1^+^ T cells, as well as Tregs, NK cells and M1 macrophages. This suggests that using MSI as the sole marker for selection of patients for immunotherapy may be insufficient. Moreover, no correlation was found in this study between MSI and CTLA-4^+^ T cells, nor between CTLA-4^+^ and PD-1^+^ T cells, suggesting that immune activity in different tumours may be regulated by different immune checkpoints. Interestingly, in an ongoing clinical trial for CRC patients combining the PD-1 inhibitor nivolumab with the CTLA-4 inhibitor ipilimumab, an improved efficiency was suggested in the combination therapy group [22]. Further studies are needed to elucidate the relative importance of CTLA-4 and PD-1 on immune cells in different molecular subgroups of CRC. 

Although the greater part of patients with MSS tumours remain unresponsive to immune checkpoint inhibitors, our findings support that selected patients with MSS CRCs (being the majority of CRCs), could potentially be candidates for immunotherapy. Our results are further supported in the literature, where some MSS tumours shown to harbour DNA polymerase ε (*POLE*) mutations and a hypermutated phenotype were shown to be enriched in neoantigens and tumour-infiltrating immune cells [23,24]. Response to PD-1 blockade by pembrolizumab has been reported in one patient with a *POLE* mutated MSS tumour [25]. Interestingly, immune profiling of MSI and *POLE* mutated tumours was shown to identify predictors of response to therapy also within these tumour subgroups [26]. Immunogenomic studies have further shown that some non-hypermutated MSS tumours actually display high neoantigen load and tumour immune infiltration, in support of the potential role of immunotherapy in these patients [27,28]. Van den Bulk et al. indeed found neoantigen-specific immunity in MSS CRCs of the CMS4 subtype [29]. Overall, these collective findings point towards the need for improved molecular tools to tailor personalised treatment plans for immunotherapy. 

Looking further into the relations of immune activation with molecular characteristics, *BRAF* mutation appeared to be associated with a slightly higher immune activity, while the opposite was found for *KRAS* mutation, associations previously suggested in literature [30,31]. Part of the association of *BRAF* mutation to the immune response may be linked to MSI, since more than 50% of *BRAF* mutated tumours are found to be MSI [32].

The more comprehensive studies of immune infiltration in CRC have used transcriptomics to evaluate the immune response in different molecular subgroups of CRC, including the gold standard CMS subgroups [18,33,34,35,36]. In brief, the four CRC CMS groups are shown to display relevant differences when it comes to immune infiltration. CMS1 and CMS4 are defined as “hot” tumours with high immune infiltration, while CMS2 and CMS3 in contrast are considered “cold” tumours with poor immune infiltration [5,33]. Even between CMS1 and CMS4, differences in the immune response have been suggested, with CMS1 being enriched in CD8^+^ cytotoxic T cells, M1 macrophages and immune checkpoint molecules such as PD-1 and CTLA-4. CMS4 tumours in contrast show a more inflamed phenotype with a high proportion of tumour promoting immune subsets, such as Tregs, myeloid-derived suppressor cells and M2 macrophages [33]. In our study, these findings are only partially reflected. For instance, we found the highest fraction of Th cells defined as Tregs in CMS1 tumours, but also in some CMS2 and CMS4 tumours. T cells were present in similar fractions in all CMS subgroups, except CMS3 tumours that displayed decreased fractions. The fraction of Tcyt cells expressing PD-1 was enriched in CMS1 tumours, but also in some CMS3 tumours. While NK cells were highly enriched in CMS1 tumours, increased fractions were also found in CMS3 tumours. Soldevilla et al. also suggested that an inflammatory immune subtype was present in CMS3 as well as CMS4 tumours [35]. In line with previous findings, macrophages were found to be slightly enriched in CMS1 tumours and were mainly of HLA-DR^+^ M1 type in this CMS subgroup. However, some CMS3 and CMS4 tumours also displayed high fractions of HLA-DR^+^ macrophages. In contrast, CMS2 tumours displayed reduced expression of HLA-DR^+^ macrophages, in line with the literature, demonstrating decreased expression of antigen presenting molecules in this subgroup [33,34]. Overall, our findings suggest that the CMS groups represent heterogenic subgroups of immune activity. Our partly contrasting findings further suggest that the groundbreaking transcriptomic analyses need to be complemented by additional studies using other methodologies. Our chosen approach of flow cytometry also comes with limitations, such as the lack of spatial context and the precision by which we managed to extract specific immune cell subsets from different tumours, which calls for further analyses using immunohistochemistry. In a study by de Vries et al. they performed comprehensive analyses of immune parameters in CRCs using both mass cytometry and multispectral fluorescence staining, revealing novel mediators of antitumour immunity [37]. A strength of our study is that we have performed very detailed descriptive molecular and immunological analyses of CRC patients. However, the study is explorative, and findings should be interpreted with some caution from a statistical point of view. The limited number of patients and the high number of statistical tests might lead to problems with multiplicity. While our findings are biologically and clinically relevant, they will require further validation in larger patient cohorts. 

A better characterisation of immune infiltration in molecular subgroups of CRC will likely assist in decisions regarding immunotherapy. One additional necessity is to find clinically relevant predictive markers. We performed comparable studies of immune activity profiles in mononuclear immune cells in blood of the corresponding patients. However, immune parameters in tumour tissue and blood did not show a strong overall relationship, suggesting that the cellular immune profile in blood is, in general, not a good mirror of the local anti-tumour immune response. Our findings are supported by transcriptomic studies, showing that markers derived from blood cells do not necessarily translate well to tissues, including tumours [38,39]. The reason for this is unclear, but may reflect a unique differentiation and activation state in the tumours. However, other cellular immune markers, as well as non-cellular systemic plasma markers could prove to be important in future therapeutic decisions. 

## 4. Materials and Methods

### 4.1. Study Cohort

Patients included were from the Uppsala-Umeå Comprehensive Cancer Consortium (U-CAN) project [40], which since 2010 longitudinally collects blood, formalin-fixed paraffin-embedded (FFPE) tissue specimens, fresh frozen tissue specimens and clinical data over time from all enrolled patients diagnosed with CRC at Umeå University Hospital, Umeå, Sweden. Clinical and pathological data was collected by one oncologist and one pathologist, respectively. Moreover, from November 2015 until July 2017, fresh tumour tissue specimens and adjacent non-malignant tissue specimens (at a distance of around 15–20 cm) were collected at the time of routine sampling at the Department of Clinical Pathology and included in the Umeå Immune Profiling of Colorectal Cancer Project (UIP-CRC). From these patients, a blood sample was also collected in the morning at the day of surgery. Rectal cancer patients who had undergone preoperative radiotherapy were excluded. Further omissions included limited tumour size where routine diagnostic sampling was prioritised instead, surgery out of laboratory hours, lack of U-CAN referral to the pathology department and other logistic problems. One-hundred and fifty-three CRC patients were included in U-CAN during the time period. Of these, 69 patients (mean age 72.8 years) were included in this study, of which immune profiles were available from tumour tissue of 64 patients, adjacent normal tissue of 57 patients and blood of 49 patients. Lack of immune profiles from some samples was the result of no available laboratory staff to handle fresh patient samples, or poor sample yield and quality. Immune profiles were also available from blood of 9 anonymous healthy donors (mean age 58.2 years). The handling of samples and patient data was approved by the Regional Ethical Review Board of Umeå, Sweden (Approval numbers: 2014/321-31 and 2016/219-31), and in accordance with the Declaration of Helsinki. All patients gave their written informed consent.

### 4.2. Isolation of Mononuclear Immune Cells

Fresh tumour and adjacent non-malignant tissue samples (5–10 mm) collected at the Department of Clinical Pathology were immediately transferred and processed at the laboratory facility for isolation of mononuclear immune cells. Tissue samples were cut into small pieces in a petri dish using a sterile scalpel. The Tumour Dissociation Kit (Miltenyi Biotec, Bergisch Gladbach, Germany) and the Octo Dissociator (Miltenyi Biotec, Bergisch Gladbach, Germany) was used for tumour tissue dissociation. In brief, disintegrated tissue was added into a gentleMACS C Tube with a volume of 5 mL enzyme mix (100 µL of Enzyme H, 50 µL of Enzyme R and 12.5 µL of Enzyme A) in RPMI1640 cell culture medium. The C tube was then placed onto the gentleMACS Octo Dissociator with Heater and the program 37C_h_TDK1 was run. After tumour tissue dissociation, isolated cells were centrifuged at 300 g for 1 min, re-suspended in RPMI and filtered through a 70 µm smart strainer (Miltenyi Biotec, Bergisch Gladbach, Germany). After washing twice, cells were re-suspended and mononuclear immune cells were isolated using Lymphoprep^TM^ (STEMCELL Technologies, Vancouver, Canada) according to manufacturer´s instructions. Fasting EDTA blood samples (10 mL) were collected in the morning prior to surgery and processed in the lab facility within two hours. For extraction of peripheral blood mononuclear cells (PBMCs), sampling tubes were centrifuged at 1400 rpm for 20 min at room temperature. The supernatant was removed, and the remaining sample was diluted with 0.9% NaCl and layered on Lymphoprep^TM^ for isolation of PBMCs as above.

### 4.3. Flow Cytometry Analyses

Flow cytometry was used to analyse the expression of immune cell surface markers. Mononuclear immune cells isolated from tumour tissue and adjacent non-malignant tissues, or blood, were washed in fluorescent activating cell sorting (FACS) medium (PBS containing 3% foetal bovine calf serum and 0.05% sodium azide), and incubated with Human TruStain FcX^TM^ (BioLegend, San Diego, CA, USA) for 10 min at room temperature to block Fc receptors. Cells were next stained using conjugated antibodies on ice for 30 min in round-bottom 96-well plates. Isotype-matched irrelevant antibodies were used as negative controls. After washing in FACS medium, staining was determined by flow cytometry (BD LSRII; BD Biosciences, San Jose, CA, USA). Information of conjugated monoclonal antibodies used can be found in Supplementary Table S2. Data was analysed with the FACSDiva software (BD Biosciences, San Jose, CA, USA) after gating on the mononuclear immune cell population in the forward scatter (FSC)/side scatter (SSC) window, followed by a gate set to identify a specific population of immune cells: T helper cells (CD3^+^/CD4^+^), cytotoxic T cells (CD3^+^/CD8^+^); monocytes/macrophages (CD14^+^); NK cells (CD56^+^/CD16^+^/CD3^−^) and B cells (CD19^+^). Doublets were eliminated based on FSC-W/FSC-H and SSC-W/SSC-H parameters. Data from 50,000 events, from the gated specific immune populations described above, was collected. The gating procedures are exemplified in Supplementary Figures S3 and S4. To evaluate the percentage of a population of gated cells expressing a specific marker (CD28, CD69, PD-1, CTLA-4, NKG2D, CD80, CD86, HLA-DR, CD163, or PD-L1), gates were set using fluorescence minus one (FMO) controls. In this study, T regulatory cells were defined as CD4^+^CD25^+^CD127^−^, previously shown to highly overlap with forkhead box P3^+^ (FOXP3^+^) Tregs [41,42].

### 4.4. Microsatellite Instability (MSI) Assessment

DNA was extracted from five FFPE sections of 10 µm from tumour and corresponding normal tissues using the AllPrep DNA/RNA FFPE kit (Qiagen, Sollentuna, Sweden), and concentration was assessed using the Qubit dsDNA BR Assay Kit (Invitrogen, Carlsbad, CA, USA). MSI analysis was performed with the MSI Analysis System Version 1.2 (Promega, Madison, WI, USA) consisting of mononucleotide repeats BAT-25, BAT-26, NR-21, NR-24, MONO-27 (and pentanucleotide repeats Penta C and Penta D for sample identification as previously described) [43]. In brief, 10 ng DNA was amplified with 1x primer mix, 1x Gold STAR Buffer (Promega, Madison, WI, USA) and AmpliTaq Gold^®^ DNA Polymerase (Applied Biosystems, Foster City, CA, USA) in a Veriti^®^ 96-Well Thermal Cycler (Applied Biosystems, Foster City, CA, USA) following the manufacturer’s instructions. PCR products were denatured in deionized formamide with Internal Lane Standard 600 (Promega, Madison, WI, USA) for allele sizing and separated using the 3130xl Genetic Analyzer. Out-data was analysed with the Peak Scanner™ Software v1.0 (Applied Biosystems, Foster City, CA, USA). The appearance of novel alleles in the tumour tissues was compared to their matched normal tissues. Tumours with two or more altered markers were classified as MSI. The remaining tumours were classified as MSS.

### 4.5. BRAF and KRAS Mutation Analyses

The *BRAFV600E* mutation was detected by digital droplet PCR (ddPCR, Bio-Rad Laboratories, Hercules, CA, USA) as previously described [43]. Briefly, a PCR sample of 20 µL was partitioned into around 20,000 nanolitre droplets. The PCR reaction was then performed in a T100 Thermal Cycler (Bio-Rad Laboratories, Hercules, CA, USA) using the program: 95 °C for 10 min; 40x cycles of 95 °C for 15 s; 56 °C for 1 min (ramp rate 2 °C/s) and 98 °C for 10 min. Then, 900 nM of the primers and 250 nM of each probe were used. The primers and probes used for detection of *BRAFV600E* were as follows: Forward: 5′-CTACTGTTTTCCTTTACTTACTACACCTCAGA-3′, reverse: 5′-ATCCAGACAACTGTTCAAACTGATG-3′, wild type probe: 5′-56-FAM/TTGGTCTAGCTACAGTGAAAT/3BHQ_1-3′ and mutation probe: 5′-5HEX/TTGGTCTAGCTACAGAGAAAT/3BHQ_1-3′ (DNA Technology A/S) [44]. The methodology behind mutational analysis of *KRAS* has been previously described [45]. In brief, *KRAS* mutations were analysed by sequencing of codon 12 and 13 using Big Dye v.3.1 (Applied Biosystems, Foster City, CA, USA). The following primers were used: Forward: 5′-TGTAAAACGACGGCCAGTGAGTTTGTATTAAAAGGTACTGG-3′ and reverse: 5′-CAGGAAACAGCTATGACCTCTGTATCAAAGAATGGTCCT-3′.

### 4.6. RNA Sequencing and Identification of Consensus Molecular Subtypes (CMSs)

RNA was extracted from a 2–3 mm cube of fresh frozen tumour tissue using the AllPrep DNA/RNA/miRNA Universal kit. Prior to extraction, tumour tissue was homogenised using a Precellys 24 homogenizer (Bertin Technologies, Montigny-le-Bretonneux, France) with 1.4 mm ceramic beads. RNA quality was assessed using Tapestation 2200 (Agilent Technologies, Santa Clara, CA, USA), and RNA concentration was measured using the Qubit dsRNA BR Assay Kit (Invitrogen, Carlsbad, CA, USA). A total amount of 1 μg RNA was used as input material for each RNA sample preparation. The RNA-sequencing library construction was performed using the NEBNext Ultra RNA Library Prep Kit for Illumina (NEB, Ipswich, MA, USA). Index codes were added to attribute sequences to each sample. Clustering of the index-coded samples was generated on a cBot Cluster Generation System using a HiSeq PE Cluster Kit cBot-HS (Illumina, Ipswich, MA, USA), according to the manufacturer’s instructions. The library preparations were next sequenced on an Illumina Hiseq platform, and 125 bp/150 bp paired-end reads were generated. Trimmed fastq files were mapped to the GRCh38 human genome assembly using HISAT2 to generate alignment files to analyse the RNA-sequencing results [46,47]. The CMS classifier for RNA-sequencing data (https://github.com/Sage-Bionetworks/crcsc) was then applied as previously described [18]. Six patients were excluded from the RNA sequencing analysis due to lack of fresh frozen tissues or poor RNA quality. Using the above-described methodology, 54 out of 63 analysed samples were classified into the CMS1-4 groups.

### 4.7. Statistics

IBM^®^ SPSS^®^ Statistics 26 (SPSS Inc, Chicago, IL, USA) was used for statistical analysis. The Mann–Whitney U test or the Kruskall–Wallis test was used to compare distributions of continuous variables between groups. For pairwise dependent continuous variables the Wilcoxon signed-rank test was performed. Correlations between continuous variables were analysed using the Spearman´s rank correlation test. The density plots were constructed using the “sm.density.compare” function from the R-package “sm” [48] in the statistical programming language R, version 4.0.0 (R Core Team, Vienna, Austria) [49]. A *p*-value < 0.05 was considered statistically significant.

## 5. Conclusions

In conclusion, our findings support the potential of using immune checkpoint inhibitor therapy in subgroups of CRC patients. We further suggest that different immune checkpoint inhibitors will likely be beneficial for selected CRC patients irrespective of MSI status, thus defining the need for improved predictive tools. Further studies are needed to understand the distribution of immune activity profiles in CRC, and to identify predictive markers that can be used to tailor personalised treatment plans.

## Figures and Tables

**Table 1:** Clinicopathological and molecular characteristics of study patients.

Characteristic	Patients, *n* (%)
**Frequency**	69 (100.0)
**Age**	
≤59	8 (11.6)
60–69	12 (17.4)
70–79	31 (44.9)
≥80	18 (26.1)
**Sex**	
Men	40 (58.0)
Women	29 (42.0)
**Stage**	
I	11 (15.9)
II	28 (40.6)
III	25 (36.2)
IV	5 (7.2)
**Tumour site**	
Right colon	37 (53.6)
Left colon	14 (20.3)
Rectum	18 (26.1)
**Tumour grade**	
Low grade	49 (71.0)
High grade	20 (29.0)
**Tumour type**	
Non-mucinous	59 (85.5)
Mucinous	10 (14.5)
***BRAF*** **mutation status**	
Wild type	45 (66.2)
Mutant	23 (33.8)
***KRAS*** **mutation status**	
Wild type	46 (71.9)
Mutant	18 (28.1)
**MSI status**	
MSS	51 (76.1)
MSI	16 (23.9)
**CMS status**	
CMS1	20 (37.0)
CMS2	23 (42.6)
CMS3	6 (11.1)
CMS4	5 (9.3)

Abbreviations: MSI, microsatellite instability, MSS, microsatellite stable; CMS, consensus molecular subtype.

**Table 2:** The correlation of expression of immune markers in tumour tissue and blood.

Immune Cell Type	Marker	*r_s_*	*p*-Value
**T helper cells**	CD4	−0.234	0.146
	CD28	0.231	0.152
	CD69	0.423	0.006
	PD-1	0.285	0.075
	CTLA-4	0.041	0.801
	Treg	0.096	0.549
**Cytotoxic T cells**	CD8	−0.161	0.314
	CD28	0.384	0.013
	CD69	−0.017	0.914
	PD-1	−0.153	0.340
	CTLA-4	0.139	0.385
	NKG2D	0.425	0.009
**NK cells**	NK	−0.191	0.226
	NKG2D	0.272	0.081
	CD69	0.231	0.140
**B cells**	CD19	0.090	0.625
	CD86	0.041	0.825
	CD80	0.322	0.072
	HLA-DR	0.105	0.567
	CD69	0.039	0.831
**Macrophages**	CD14	−0.058	0.724
	HLA-DR	−0.305	0.059
	CD163	0.038	0.820
	PD-L1	0.078	0.636

r_s_, Spearman’s rank correlation coefficient.

## Supplementary Materials

The following are available online at https://www.mdpi.com/2072-6694/12/11/3440/s1, Figure S1: Distribution of immune profiles in MSI/MSS subgroups, Figure S2: Distribution of immune profiles in CMS subgroups, Figure S3: Gating procedures for lymphocyte subsets, Figure S4: Gating procedures for innate immune cells, Table S1: Correlations of immune profiles in tumour tissue and blood, Table S2: Antibodies used for flow cytometry analyses.
