# Supplementary material for: A Detailed Flow Cytometric Analysis of Immune Activity Profiles in Molecular Subtypes of Colorectal Cancer
Source: Cancers (Basel). 2020 Nov 19;12(11):3440. doi: 10.3390/cancers12113440 (PMC7699331; doi:10.3390/cancers12113440)
Supplement: Supplementary file 1 [file cancers-12-03440-s001.pdf]

cancers

MDPI

Supplementary materials: A Detailed Flow Cytometric Analysis of Immune Activity Profiles in Molecular Subtypes of Colorectal Cancer

Xingru Li, Agnes Ling, Therese G. Kellgren, Marie Lundholm, Anna Löfgren-Burström, Carl Zingmark, Martin Rutegård, Ingrid Ljuslinder, Richard Palmqvist and Sofia Edin

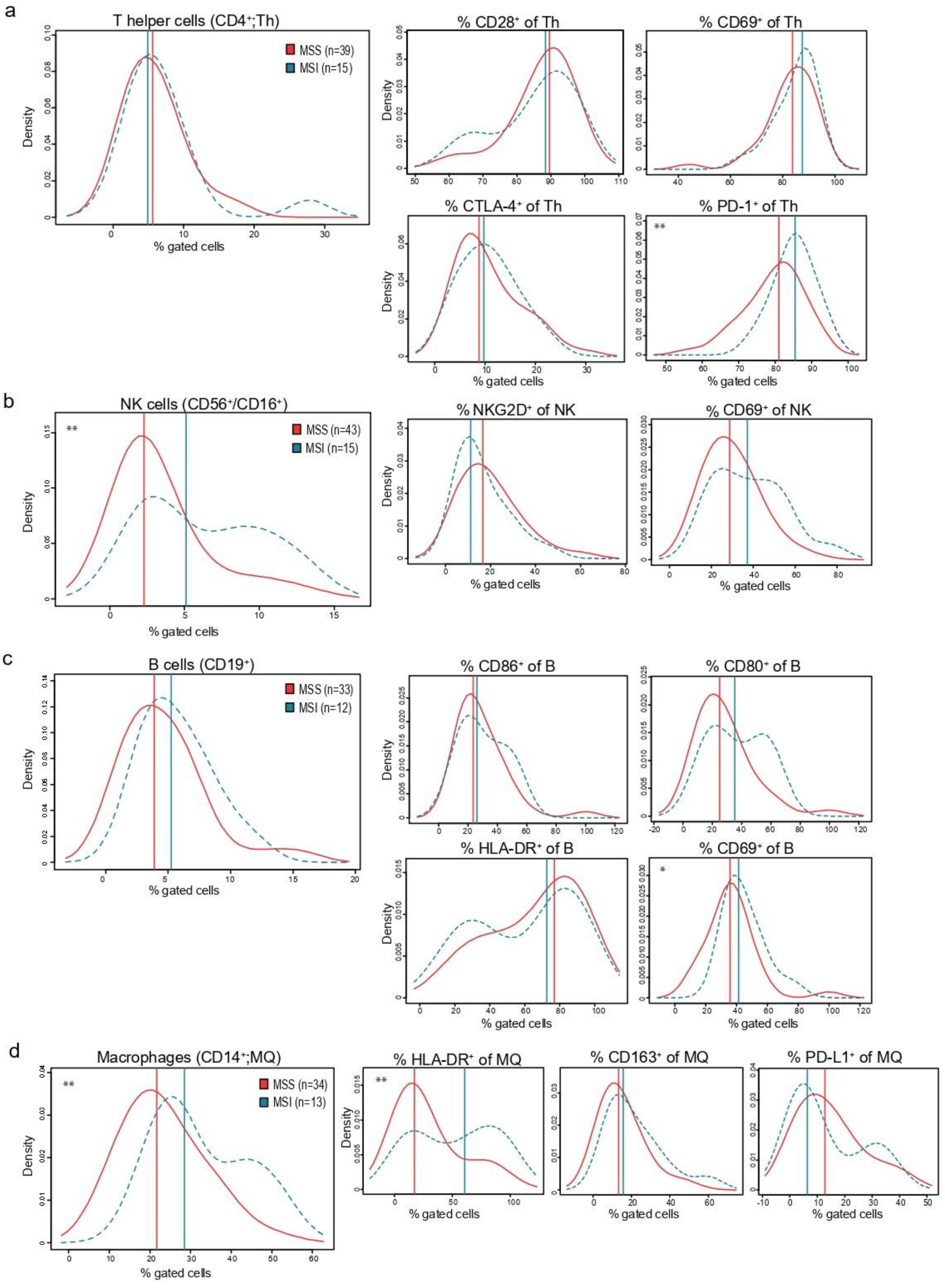

Figure S1. Distribution of immune profiles in MSI/MSS subgroups. Density plots illustrating the distribution of immune markers for; (**a**) T helper cell subsets, (**b**) NK cell subsets, (**c**) B cell subsets and (**d**) macrophage subsets predicted by MSI/MSS subgroups. Vertical lines indicate median values. *indicate P-values <0.1 and **indicate P-values < 0.05.

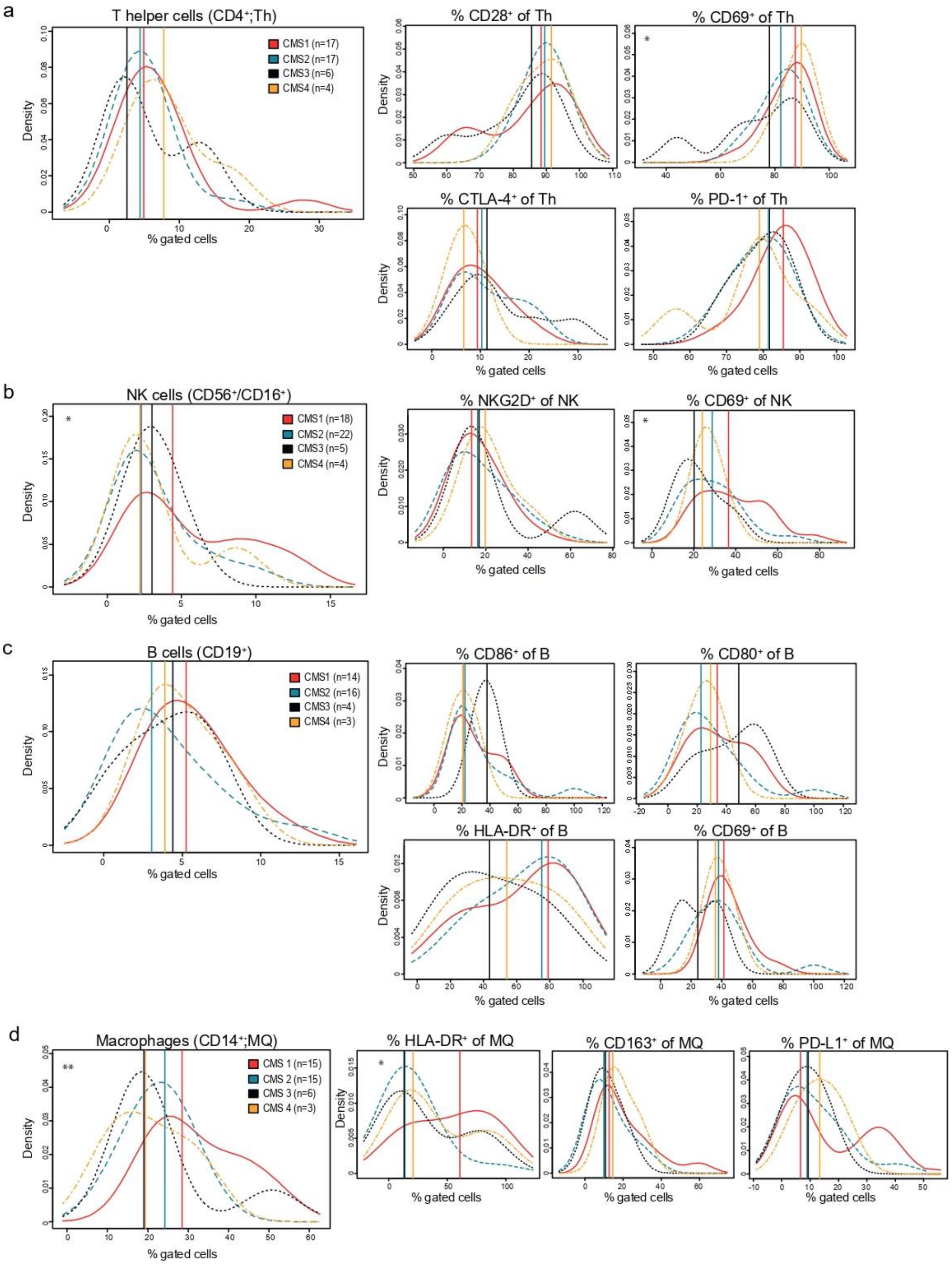

Figure S2. Distribution of immune profiles in CMS subgroups. Density plots illustrating the distribution of immune markers for; (**a**) T helper cell subsets, (**b**) NK cell subsets, (**c**) B cell subsets and (**d**) macrophage subsets predicted by CMS subgroups. Vertical lines indicate median values. *indicate *P*-values < 0.1 and **indicate *P*-values < 0.05.

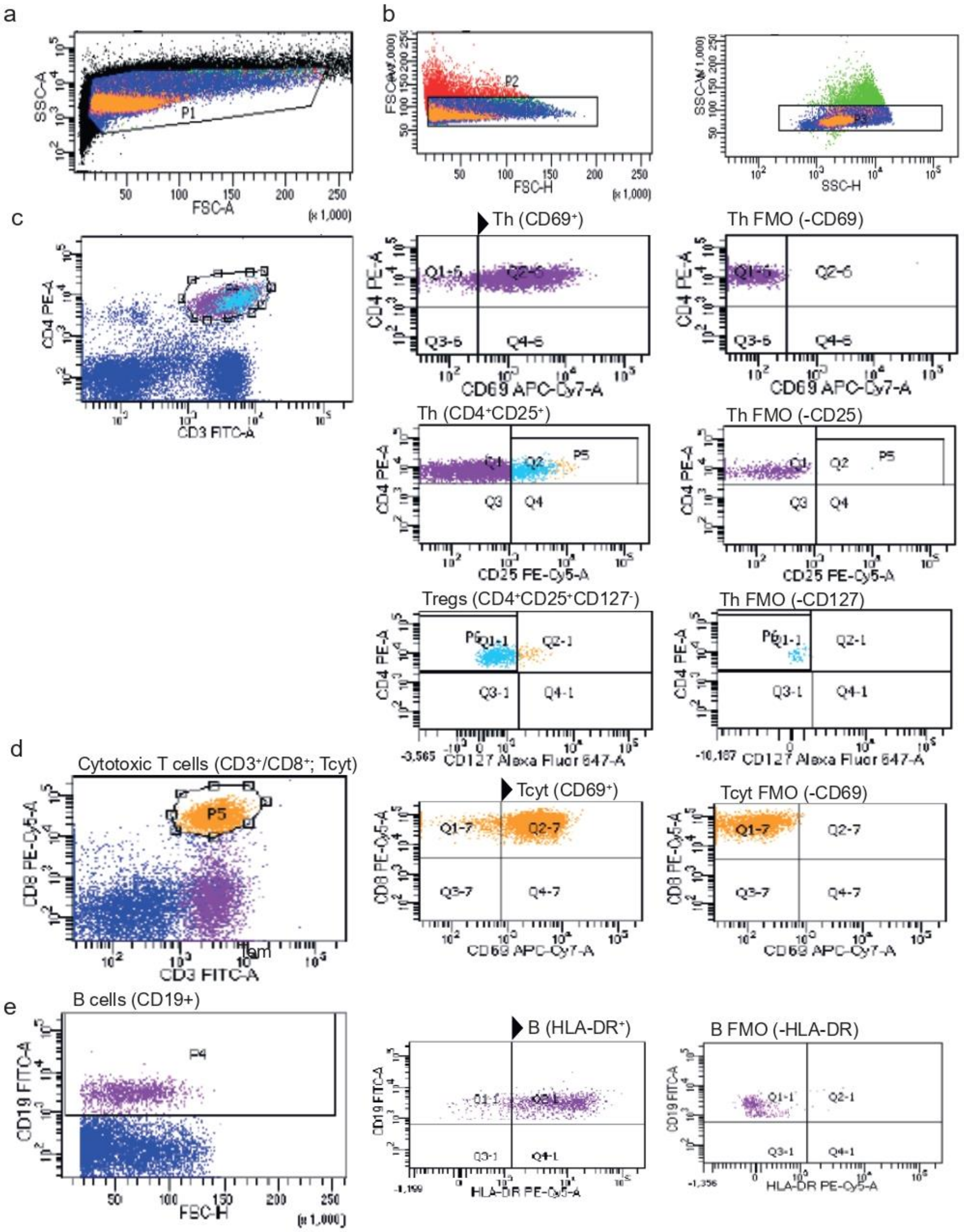

Figure S3. Gating procedures for lymphocyte subsets. The gating procedures are exemplified using the indicated immune markers. An initial gate was set in the FSC/SCC window to identify mononuclear immune cells (a), followed by gating to eliminate doublets based on FSC-W/FSC-H and SSC-W/SSC-H parameters (b). A gate was next set to identify a specific population of lymphocytes; (c) T helper cells ($CD3^+/CD4^+$), (d) cytotoxic T cells ($CD3^+/CD8^+$), and (e) B cells ($CD19^+$). To evaluate the percentage of a population of gated cells expressing a specific marker as indicated, gates were set using FMOs (flourescence minus one) controls. T regulatory cells were defined as $CD4^+CD25^+CD127^-$.

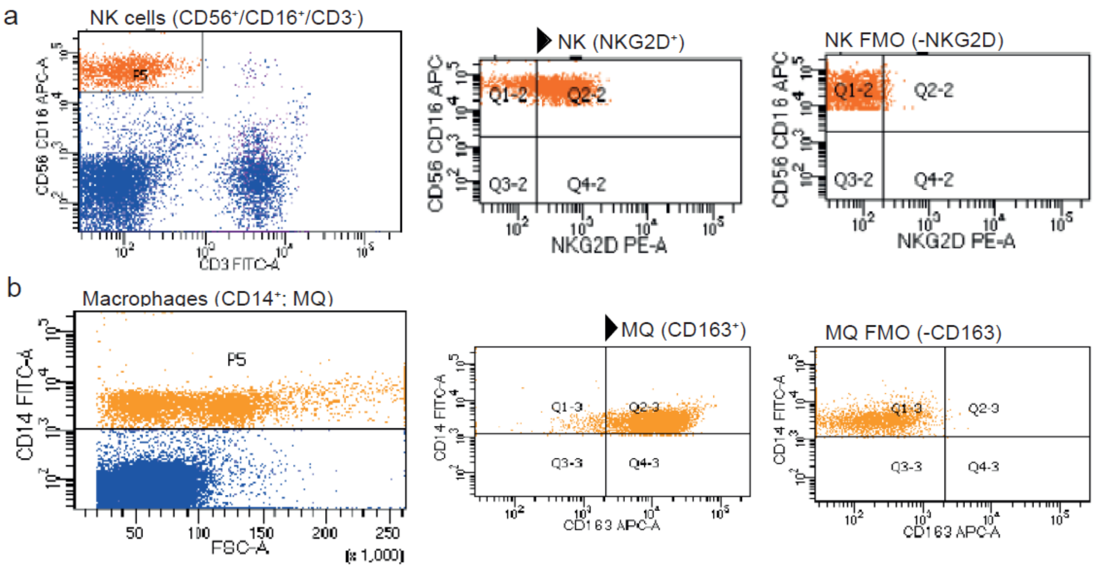

Figure S4. Gating procedures for innate immune cells. The gating procedures are exemplified using the indicated immune markers. A gate was first set to identify a specific population of innate immune cells within the mononuclear immune cell population; (**a**) NK cells ($CD56^+/CD16^+/CD3^-$), and (**b**) monocytes/macrophages ($CD14^+$). To evaluate the percentage of a population of gated cells expressing a specific marker as indicated, gates were set using FMOs (flourescence minus one) controls.

Table S1. Correlations of immune profiles in tumour tissue and blood.

		Blood																							
		T helper cells						Cytotoxic T cells						NK cells			B cells					Macrophages			
**Tumour tissue**		**CD4**	CD28	CD69	PD1	CTLA4	Treg	**CD8**	CD28	CD69	PD1	CTLA4	NKG2D	**NK**	NKG2D	CD69	**CD19**	CD86	CD80	HLADR	CD69	**CD14**	HLADR	CD163	PDL1
**T helper cells**	**CD4**	-0.234	0.055	-0.325*	-0.057	0.192	-0.118	-0.232	-0.014	-0.307	-0.386*	0.196	-0.337*	0.099	-0.253	-0.344*	-0.334*	-0.039	-0.031	-0.247	-0.242	-0.244	-0.271	-0.220	0.096
	CD28	0.396*	0.231	0.189	0.022	0.279	0.275	0.190	0.099	0.203	0.235	0.351*	0.020	-0.090	-0.018	0.291	0.259	0.210	0.236	0.135	0.155	0.325*	0.089	-0.236	-0.103
	CD69	0.082	-0.173	0.423**	0.208	-0.155	0.168	0.373*	-0.025	0.188	0.191	0.17	0.144	-0.092	0.088	0.229	0.023	-0.027	0.016	0.130	0.191	0.206	0.103	0.245	-0.284
	PD1	0.030	-0.191	0.079	0.285	-0.263	0.106	0.102	-0.147	0.025	0.166	0.069	0.228	0.032	0.087	-0.058	-0.128	0.27	0.242	0.051	0.242	0.056	0.102	0.112	-0.043
	CTLA4	0.335*	0.311	-0.203	-0.069	0.041	0.244	-0.070	0.040	-0.103	0.081	0.188	0.133	0.046	0.126	0.016	0.161	0.130	0.233	0.083	0.125	0.121	-0.049	-0.207	0.091
	Treg	-0.228	-0.099	-0.014	0.233	0.147	0.096	0.146	-0.082	-0.078	-0.103	0.297	0.158	0.151	0.078	0.040	-0.159	-0.034	0.035	0.174	-0.166	0.039	0.323*	-0.164	0.183
**Cytotoxic T cells**	**CD8**	-0.316*	-0.173	-0.262	0.065	0.100	-0.198	-0.161	-0.215	-0.330*	-0.273	0.077	-0.297	0.074	-0.344*	-0.472**	-0.281	0.017	-0.068	-0.313	-0.124	-0.195	-0.291	-0.148	-0.105
	CD28	-0.185	0.170	0.278	0.12	0.072	0.07	0.105	0.384*	0.339*	0.164	-0.141	-0.147	0.115	-0.148	0.182	0.091	0.018	0.193	0.171	0.061	-0.005	0.240	0.011	0.133
	CD69	-0.029	-0.279	-0.029	0.089	-0.009	0.128	-0.021	-0.163	-0.017	-0.113	0.037	0.032	0.005	-0.052	-0.108	-0.163	0.137	0.173	0.011	0.088	0.030	0.146	-0.004	-0.003
	PD1	-0.017	-0.055	-0.275	-0.037	-0.222	0.005	-0.221	-0.029	-0.310*	-0.153	-0.030	-0.020	-0.059	-0.138	-0.344*	-0.232	-0.025	-0.021	-0.077	0.048	-0.166	-0.187	-0.039	-0.078
	CTLA4	0.267	0.391*	0.017	-0.167	0.225	0.216	-0.100	0.103	-0.004	0.013	0.139	0.053	-0.002	0.121	0	0.287	0.074	0.174	0.050	0.096	0.069	-0.195	-0.138	-0.034
	NKG2D	0.077	0.013	0.217	0.113	0.059	0.223	0.259	0.108	0.189	0.229	0.285	0.425**	-0.198	0.319	0.491**	0.030	0.155	0.146	-0.085	0.047	0.202	0.220	0.147	-0.043
**NK cells**	**NK**	-0.163	0.007	-0.409*	-0.091	0.033	0.015	-0.243	-0.201	-0.298	-0.292	0.183	0.124	-0.191	0.143	-0.235	-0.217	0.049	0.014	-0.188	-0.067	-0.277	-0.101	-0.284	0.277
	NKG2D	0.079	-0.162	0.330*	0.160	-0.276	0.129	0.244	0.064	0.141	0.146	-0.230	-0.147	0.138	0.272	0.217	0.084	0.341*	0.194	-0.19	0.175	0.054	-0.005	0.402**	-0.088
	CD69	0.158	-0.081	0.339*	0.141	-0.138	0.264	0.369*	0.107	0.082	0.008	0.048	-0.160	0.316*	0.170	0.231	0.157	0.177	-0.024	0.274	0.226	0.276	0.129	0.437**	-0.009
**B cells**	**CD19**	-0.01	-0.049	0.068	0.018	0.170	0.253	0.017	-0.081	-0.059	-0.212	0.235	0.044	-0.076	0.208	0.072	0.09	0.022	0.104	-0.232	0.074	-0.181	-0.133	-0.039	0.125
	CD86	0.081	0.016	-0.099	0.115	-0.097	0.148	0.252	0.043	-0.110	0.002	0.269	0.137	-0.052	0.385*	0.165	-0.108	0.041	0.055	0.154	0.040	0.021	0.161	0.217	0.124
	CD80	0.101	-0.115	-0.056	0.269	0.058	0.372*	0.179	0.035	-0.009	0.114	0.191	0.176	0.254	0.294	0.118	0.015	0.088	0.322	0.212	0.276	0.155	0.129	0.203	0.343
	HLADR	-0.135	0.130	0.001	-0.15	0.051	-0.114	-0.223	0.118	-0.021	-0.158	-0.196	-0.348	0.193	-0.316	-0.251	-0.020	-0.181	-0.177	0.105	-0.236	-0.174	-0.042	-0.151	0.019
	CD69	-0.185	-0.140	0.006	-0.032	0.070	0.042	0.143	-0.04	-0.146	-0.305	0.246	0.232	0.095	0.202	0.086	-0.065	0.031	0.069	-0.044	0.039	0.009	0.093	0.084	0.106
**Macrophages**	**CD14**	-0.033	-0.283	0.053	0.076	0.170	-0.057	0.252	-0.039	0.051	-0.010	0.137	0.037	0.093	-0.061	0.209	-0.049	0.179	0.014	0.044	0.096	-0.058	0.295	0.095	0.111
	HLADR	-0.152	-0.084	-0.264	-0.078	-0.109	-0.132	-0.262	-0.195	-0.380*	-0.315	-0.176	-0.259	0.057	-0.212	-0.468**	-0.392*	-0.080	0.031	-0.196	-0.095	-0.293	-0.305	0.01	-0.084
	CD163	-0.013	-0.233	-0.057	0.258	0.159	-0.013	0.107	-0.309	-0.140	0.056	0.328	0.190	0.235	0.117	-0.100	0.142	0.031	0.271	-0.275	-0.026	-0.086	0.037	0.038	0.056
	PDL1	0.199	0.071	0.052	-0.112	-0.002	0.032	-0.233	-0.098	0.079	-0.123	-0.141	-0.191	0.424**	-0.088	-0.219	0.135	0.011	0.106	0.009	-0.030	-0.081	0.010	-0.003	0.078

Shown is r_s, Spearmans rank correlation coefficient.

(yellow) $P < 0.05$

(red) $P < 0.01$

Table S2. Antibodies used for flow cytometry analyses.

Marker	Flourochrome	Clone	Clonality	Company
CD3	FITC	UCHT-1	monoclonal	R&D systems
CD4	PE	RPAT4	monoclonal	BD Biosiences
CD25	PE-Cy5	M-A251	monoclonal	BD Biosiences
CD127	AlexaFluor 647	HIL-7R-M21	monoclonal	BD Biosiences
CTLA-4 (CD152)	Brilliant violet 421	BNI3	monoclonal	BD Biosiences
NKG2D	PE	149810	monoclonal	R&D systems
CD8	PE-Cy5	RPA-T8	monoclonal	BD Biosiences
CD16	APC	3G8	monoclonal	Life Technologies
CD56	APC	B159	monoclonal	BD Biosiences
CD69	APC-Cy7	FN50	monoclonal	BD Biosiences
CD28	APC	CD28.2	monoclonal	BD Biosiences
CD14	FITC	M5E2	monoclonal	BD Biosiences
CD86	PE	-	monoclonal	BD Biosiences
HLA-DR	PE-Cy5	G46-6	monoclonal	BD Biosiences
CD163	Alexa Fluor 647	GHI/61	monoclonal	BD Biosiences
CD80	PE-Cy7	L307.4	monoclonal	BD Biosiences
CD19	FITC	HIB19	monoclonal	BD Biosiences
PD-1 (CD279)	PE-Cy7	EH12.1	monoclonal	BD Biosiences
PD-L1	BV421	MIH1	monoclonal	BD Biosiences
